# PbrMYB14 Enhances Pear Resistance to *Alternaria alternata* by Regulating Genes in Lignin and Salicylic Acid Biosynthesis Pathways

**DOI:** 10.3390/ijms26030972

**Published:** 2025-01-24

**Authors:** Qi Yan, Weiyi Chen, Hui Zhang, Peng Liu, Yuxing Zhang

**Affiliations:** 1 College of Horticulture, Hebei Agricultural University, Baoding 071000, China; yanqi19938@163.com (Q.Y.); zhanghui08020212@163.com (H.Z.); lp3752103@163.com (P.L.); 2 College of Agronomy, Hebei Agricultural University, Baoding 071000, China; cwy2232199420@163.com

**Keywords:** plant resistance, transcriptional control, plant immunity, plant phenylalanine metabolism, plant immunity enhancers, pear breeding

## Abstract

Pear is an important originate fruiter in China, ranking first in the world in terms of cultivation area and yield. However, it is susceptible to infection by *Alternaria alternata* (*A. alternata*), resulting in a reduction of approximately 30% in yield. While both lignin and salicylic acid (SA) are recognized as key components of plant immune responses, the molecular mechanisms connecting these pathways remain poorly understood. Here, we have discovered a nuclear localization transcription activator PbrMYB14 in pears, whose expression can be induced by exogenous SA and *A. alternata*. Overexpression of *PbrMYB14* significantly increased lignin and SA content in pears, making them more resistant to *A. alternata*, and the relative lesion area decreased by 68.95% compared with WT plants. By analyzing the transcriptome of *PbrMYB14*-overexpressing plants, the lignin synthesis gene *Pbr4CL1* and SA synthesis gene *PbrPAL1* regulated by PbrMYB14 were screened and identified. Through yeast one-hybrid (Y1H) and a Dual-Luciferase assay (LUC), it was confirmed that PbrMYB14 positively regulates the expression of *Pbr4CL1* and *PbrPAL1* genes. Our results suggest that PbrMYB14 links lignin resistance and SA resistance in pears, providing valuable information for future genetic breeding research on pear disease resistance.

## 1. Introduction

Pear (*Pyrus* spp.) is a member of the family Rosaceae with a long cultivation history and extensive germplasm resources, and it is cultivated in all temperate regions of the world. It is one of the most economically important fruit commodities throughout the world, particularly in China [1,2]. ‘Duli’ (*Pyrus betulifolia* Bunge) has a well-developed root system and strong stress resistance, making it an ideal material for transgenic research [3]. However, they are highly susceptible to pathogenic fungal infections during growing season, harvest, and storage [4]. Pear black spot (PBS) is a common disease during the growth period of pear trees, and it is also an important disease during the storage period of pear fruits, seriously affecting the yield and quality of pears [5]. The optimal spore germination temperature for *A. alternata* is 28 °C, and the relative humidity is within the range of 50% to 100%. Therefore, appropriate conditions such as high temperature and high humidity make pear black spot disease happen more seriously and severely [6]. At present, the method of preventing and controlling pear black spot disease is to use chemical agents such as sulfur compounds and manganese zinc [7]. However, long-term use of chemical agents cause pathogens to develop resistance, harm human health, and cause environmental pollution [8]. In addition, the number and variety of pear varieties resistant to black spot disease are limited, which cannot meet the needs of different regions and planting conditions. Consequently, it is particularly necessary to cultivate disease resistant varieties through genetic improvement.

Previous studies have shown that polyphenolic compounds of the phenylpropanoid pathway, such as lignin and SA, play important roles in plant disease resistance [9,10]. SA is an important plant hormone that activates plant defense responses through signal transduction and expression of disease-related protein proteins, thickening and lignification of plant cell walls, induction of systemic acquired resistance (SAR), and interaction with other signaling molecules, thereby enhancing plant resistance to pathogens [11]. Lignin, as one of the main components of the plant cell wall, plays an important role in determining plant cell wall mechanical strength, rigidity, and hydrophobic properties. Enhancing the mechanical strength and stiffness of cell walls provides a physical barrier for plants to resist the invasion and spread of pathogens; the hydrophobic properties of cell walls affect the adsorption capacity and colonization efficiency of pathogens and trigger immune responses to resist pathogen invasion [12]. Lignin deficiency leads to reduced growth and dwarfing of phenylpropanoid mutants [13]. Due to the complexity of its structure, lignin itself is difficult to degrade, so it plays a role in protecting polysaccharides such as cellulose from degradation by microorganisms and enzymes during plant cell growth. This characteristic gives lignin the ability to resist external mechanical damage and the invasion and spread of plant pathogens [14]. For example, in eucalyptus, the deposition of lignin in necrotic periderm in the early stages of infection by *Mycosphaerella* explains the greater resistance of *Eucalyptus nitens* as compared with *E. globulus* [15]. Comparative metabolite profiling of xylem tissue of *Ulmus minor* and *U. minor* × *U. pumila* after inoculation with *Ophiostoma novo-ulmi* showed that the hybrid has a faster defense response, which was characterized by an increase in the amount of lignin [16]. There are reports that salicylic acid increases plant resistance to pathogens by promoting lignin synthesis. The transcription factor WRKY45 related to disease resistance and the lignin synthesis gene was simultaneously induced to express by SA, and the induced expression of the lignin synthesis gene lagged behind the expression of *WRKY45* [17]. While both lignin and SA are recognized as key components of plant immune responses, the molecular mechanisms connecting these pathways remain poorly understood.

The MYB gene family has been reported to be one of the largest transcription factor (TF) families in the plant kingdom [18]. MYB TFs act as key regulators and affect plant growth and resistance by regulating target genes related to substance synthesis and metabolism [19]. MYB proteins are characterized by a highly conserved MYB domain, and each consists of about 50 amino acids and a helix-turn-helix structure [20]. MYB TFs are classified into four subfamilies, R2R3-MYB,1R-MYB, 3R-MYB, and 4R-MYB factors, depending on the number of adjacent repeats (R1, R2, and R3) in the DNA-binding domain. According to reports, the *Arabidopsis thaliana* SG2-type R2R3-MYB TF MYB15 is a regulator of defense-induced lignification and basal immunity [21]. OsMYB30 bound and activated the promoters of 4-coumarate: CoA ligase genes (*Os4CL3/5*), resulting in the accumulation of lignin subunits G and S. This action led to obvious thickening of sclerenchyma cells near the epidermis, inhibiting *M. oryzae* penetration at the early stage of infection [22]. GhMYB18 was involved in the defense response to cotton aphid by participating in the synthesis of SA and flavonoids [23]. MdMYB73 confers increased resistance to the fungal pathogen *Botryosphaeria dothidea* in apples via the SA pathway [24]. MYB96-mediated ABA signals enhance plant disease resistance by inducing SA biosynthesis [25]. MYB115 contributes to the regulation of proanthocyanidin biosynthesis, which could be effectively employed for the metabolic engineering of proanthocyanidins (PAs) to improve resistance to fungal pathogens [26]. VqMYB154 from *V. quinquangularis* accession Danfeng-2 participates in the regulatory mechanism that improves the biosynthesis and accumulation of stilbenes and enhances resistance to disease in grapevine [27]. Brown planthopper (BPH) feeding induces the expression of an R2R3-MYB TF, which in turn upregulates the expression of *OsPALs* genes; these genes encode phenylalanine ammonia-lyase enzymes, essential for the phenylpropanoid pathway, which drives the production of defense-related metabolites like lignin and salicylic acid, thereby enhancing rice resistance to brown planthoppers [10]. OsMYB30, OsMYB55, and OsMYB110 are involved in the signal pathway between MAMP perception and cinnamate/monolignol synthesis and have important roles for plant immunity [28].

In the present study, transgenic ‘Duli’ with *PbrMYB14*-overexpressing plants was generated. The *A. alternata* resistance of the transgenic plants were investigated, and RNA sequencing (RNA-Seq) was carried out to assess its downstream transcriptional effects. Moreover, Y1H and LUC assays were used to characterize the regulation of *Pbr4CL1* and *PbrPAL1* by PbrMYB14. Could the dual regulation of lignin and SA pathways by PbrMYB14 pave the way for breakthrough strategies in crop protection?

## 2. Results

### 2.1. Effect of A. alternata on Pear Leaves

**Figure 1:**
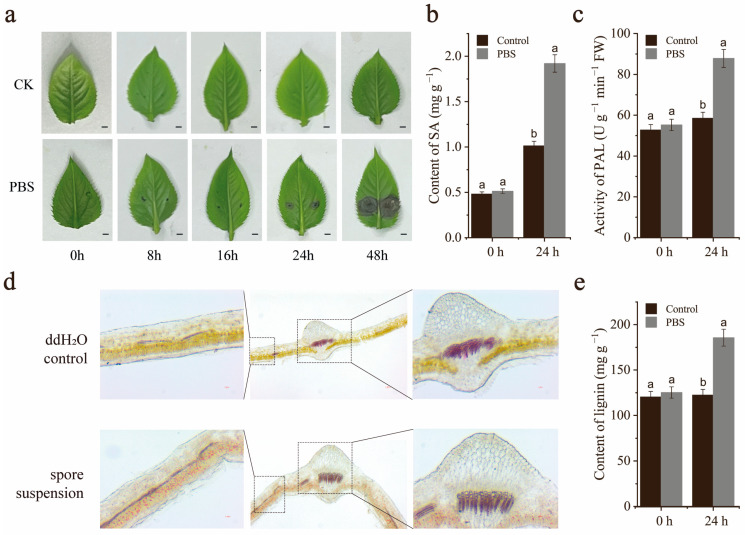
Effect of *A. alternata* on pear leaves. (**a**) Phenotypic changes within 48 h after inoculation with *A. alternata* on the leaves of ‘Xinli No. 7’. Bar = 1 mm. (**b**) High-performance liquid chromatography (HPLC) was used to detect the content of SA. (**c**) Activity of phenylalanine ammonia lyase. (**d**) Lignin analysis of leaves stained with phloroglucinol-HCl. Stained leaves induced by *A. alternata* for 24 h. (**e**) The AcBr method is used to detect lignin content. PBS, inoculated with *A. alternata*. The data represent the mean ± standard error (*n* = 3) of three biological replicates. The error bars represent the SDs obtained from three replicates. Bars with the same letter represent no significant differences (*p* < 0.05).

PBS is a common disease during the growth period of pear trees, and it is also an important disease during the storage period of pear fruits, seriously affecting the yield and quality of pears. In this study, we investigated the effect of *A. alternata* on pear leaves. The leaves of the test tube seedlings of ‘Xinli No. 7’ (*Pyrus sinkiangensis*) began to show circular black spots around 24 h after inoculation with *A. alternata*, and the spots gradually expanded and turned black-brown (Figure 1a).

The preliminary research results in the laboratory showed that the SA content significantly increased after inoculation with *A. alternata*. Therefore, we tested that the SA content in the leaves was significantly higher than the control after 24 h of inoculation with *A. alternata* (Figure 1b). As phloroglucinol-HCl staining revealed qualitative differences in lignification between leaves inoculated with pathogens and not inoculated, we further measured lignin content of the leaves using the acetyl bromide (AcBr) method. Consistent with our phloroglucinol-HCl staining results, PBS treatment significantly increased the lignin content in leaves (Figure 1d,e). Due to the ability of lignin and SA to be synthesized through phenylalanine, we tested the phenylalanine ammonia lyase activity in leaves inoculated with *A. alternata* (Figure 1c) (*p* < 0.05). The results showed that the phenylalanine ammonia lyase activity in leaves inoculated with PBS was significantly stronger than that in the control.

### 2.2. Cloning and Sequence Analysis of PbrMYB14

The genomic DNA and cDNA of ‘Xinli No. 7’ were used as templates for amplification, and sequences with lengths of 1990 and 801 bp were obtained, respectively. *PbrMYB14* was located on chromosome 16. By comparing genomic DNA with cDNA sequences, *PbrMYB14* was found to contain three exons and two introns (Figure S1a). The CDS of *PbrMYB14* encodes a 267 amino acid protein.

Multiple comparative analyses indicated that PbrMYB14 is an SG2-type R2R3-MYB TF, containing a highly conserved pair of SG2 motifs in its C-terminal region (Figure S1b). A phylogenetic tree of PbrMYB14 and *Arabidopsis* MYB family genes was constructed by referring to the standard classification of *Arabidopsis* MYB members to carry out phylogenetic analysis and clustering. Phylogenetic analysis and sequence alignment reveal that PbrMYB14 is closely related to the SG2-type R2R3-MYB TF AtMYB15, which has been reported to regulate lignin biosynthesis (Figure S1c).

### 2.3. Analysis of the Subcellular Localization and Transcriptional Activity of PbrMYB14

**Figure 2:**
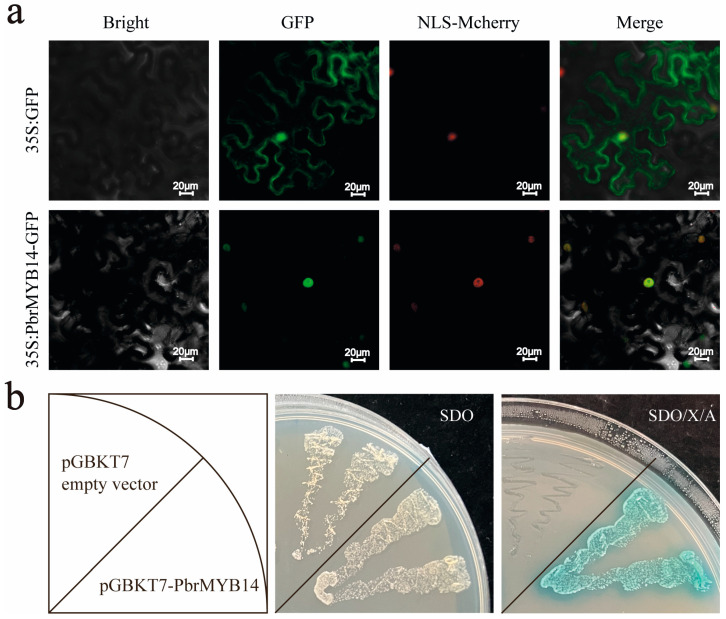
Subcellular localization and transcriptional activity assays of PbrMYB14. (**a**) PbrMYB14 is localized in the nucleus based on transient expression in tobacco leaves. Bar = 20 μm. (**b**) Transcriptional activation of PbrMYB14 in yeast. The yeast strain Y2HGold carrying GAL4BD-PbrMYB14 and the pGBKT7 empty vector was cultured on synthetic dropout medium without tryptophan (SDO) or SDO medium supplemented with X-α-gal and AbA (SDO/X/A) at 30 °C for 3 days.

In order to explore the subcellular localization characteristics of PbrMYB14, a vector containing the fusion between the PbrMYB14 coding sequence and *GFP* gene under the control of the cauliflower mosaic virus (CaMV) *35S* promoter and a control vector including the *35S:*GFP cassette were constructed. Both the fusion vector and the control vectors were transiently introduced into the tobacco leaves. Microscopic observation showed that the GFP signal of the control was observed throughout the nucleus and cytoplasmic membrane, while the green fluorescence signal of the *PbrMYB14*-GFP fusion was observed exclusively in the nucleus (Figure 2a). The results indicated that PbrMYB14 is a nuclear protein.

To determine whether PbrMYB14 has transcriptional activation, we fused the full-length *PbrMYB14* downstream of the GAL4 DNA-binding domain (GAL4BD) in the pGBKT7 vector and transformed it into Y2HGold yeast. All yeast cells grew normally on SDO, while only cells transformed with the GAL4BD-PbrMYB14 vector were survived on SDO/X/A and appeared blue when cultured on selective media (Figure 2b). These results indicate that the GAL4BD-PbrMYB14 fusion protein activates the expression of reporter genes *MEL1* and *AUR1*, indicating that PbrMYB14 has transcriptional activation potential. These results indicate that PbrMYB14 is a transcription activator located in the nucleus.

### 2.4. PbrMYB14 Was Positively Induced by A. alternata

We further studied the expression patterns of *PbrMYB14* after infection with *A. alternata*. The expression of *PbrMYB14* increased sharply and peaked at 16 h after infection with *A. alternata* at a level that was 6.48 times higher than that of the control. Thereafter, the expression level remained higher than that of the control group until 48 h, showing an overall upward trend (Figure S2). The results showed that PbrMYB14 was positively expressed during infection with *A. alternata*.

### 2.5. Overexpression of PbrMYB14 Increased the Transcript Abundance of Endogenous

**Figure 3:**
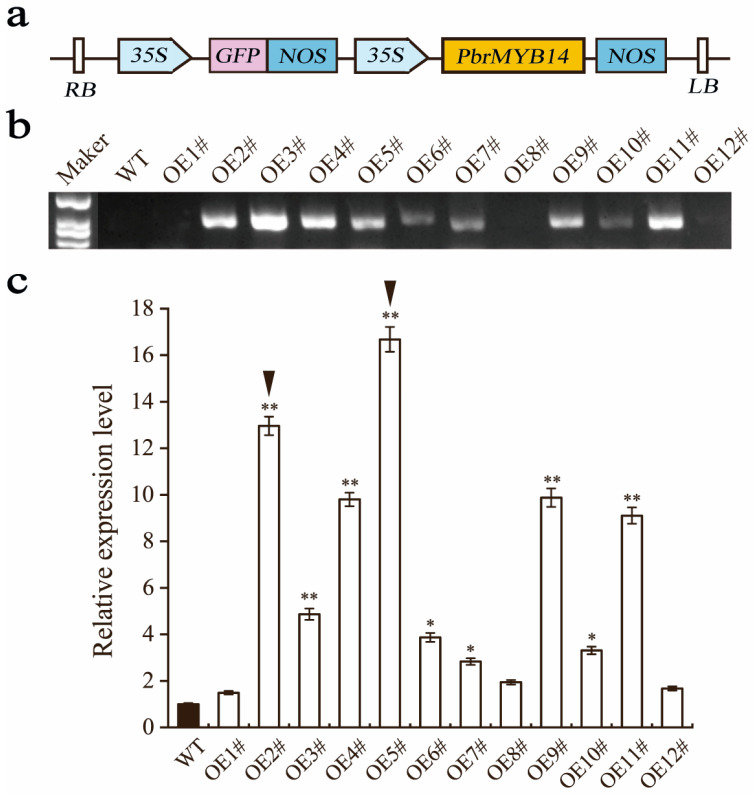
Identification of *PbrMYB14*-overexpression plants. (**a**) Schematic diagram of overexpression recombinant vector construction. *35S*, *CaMV35S* promoter; GFP, green fluorescent protein; NOS, NOS terminator; RB, right boundary; LB, left boundary. (**b**) PCR identification of *PbrMYB14*-overexpressing plants. (**c**) The expression level of *PbrMYB14* gene in *PbrMYB14*-overexpression plants. The black triangle represents that we selected these two overexpression lines for subsequent experiments. The error bars represent the standard deviation (SD) of three biological replicates. Asterisks indicate significant differences (* *p* < 0.05, ** *p* < 0.01; based on the *t*-test).

In order to understand the mechanism of PbrMYB14 in response to infection with *A. alternata*, we overexpressed it via the *Agrobacterium*-mediated transformation system of ‘Duli’ cotyledons, which further evaluated the function of PbrMYB14. PCR detection was performed using DNA from *PbrMYB14*-overexpressing plants as a template. The results showed that 9 out of 12 overexpression lines could amplify a band of 885 bp (*35S:PrMYB14*) (Figure 3a,b). The results indicate that *35S:PrMYB14* has been successfully inserted and expressed in the *PbrMYB14*-overexpressing plants.

Collect leaves from *PbrMYB14*-overexpression plants and extract RNA. The expression of *PbrMYB14* gene in the *PbrMYB14*-overexpression plants were detected by qRT-PCR (Figure 3c). The results showed that the expression of *PbrMYB14* in nine *PbrMYB14*-overexpression plants were significantly higher than that in wild-type (WT) plants. These results indicate that we have successfully obtained *PbrMYB14*-overexpression plants.

### 2.6. Overexpression of PbrMYB14 Affects SA and Lignin Content

**Figure 4:**
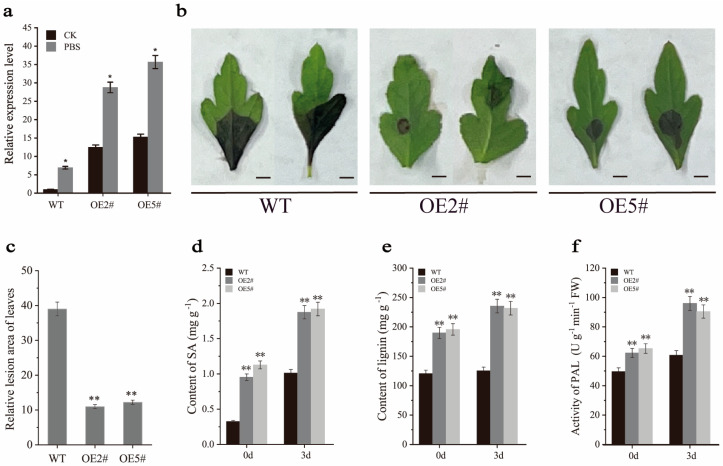
Evaluation of resistance to *A. alternata* in the *PbrMYB14*-overexpressing plants and WT plants. (**a**) Expression of the *PbrMYB14* gene in *PbrMYB14*-overexpressing plants after inoculation with *A. alternata*. (**b**) Phenotypic after inoculation for 3 days with *A. alternata* on the leaves of the *PbrMYB14*-overexpressing plants and the WT plants. (**c**) The relative lesion areas of the *PbrMYB14*-overexpressing plants and WT plants. Bar = 1 mm (**d**) HPLC was used to detect the content of SA. (**e**) The AcBr method was used to detect lignin content. (**f**) Activity of phenylalanine ammonia lyase. We measured these indicators 3 days after inoculation. The error bars represent the standard deviation (SD) of three biological replicates. Asterisks indicate significant differences (* *p* < 0.05, ** *p* < 0.01; based on *t*-test).

In order to investigate whether PbrMYB14 is involved in the defense of pear against *A. alternata*, we selected two highly expressed transgenic lines OE#2 and OE#5 for inoculation with *A. alternata* based on the expression level of the transgene, and WT plants and transgenic plants were selected for pathogen infection test. The expression of *PbrMYB14* in *PbrMYB14*-overexpressing plants after infection with *A. alternata* was significantly higher than WT plants (Figure 4a), which was characterized by severe spread of gray black lesions and scorched necrosis (Figure 4b). After 3 days of infection, the relative lesion areas of WT, OE#2, and OE#5 were 39.02%, 10.99%, and 12.24%, respectively. The relative lesion area of the leaves of *PbrMYB14*-overexpressing plants were significantly lower than that of the WT plants (Figure 4c), which were 28.03% and 26.78% lower than the WT plants, respectively.

In order to further investigate whether the disease resistance of PbrMYB14 is related to SA and lignin, firstly, we tested the SA content in leaves and found that the SA content in the *PbrMYB14*-overexpressing plants was significantly higher than that in control. After inoculation with *A. alternata*, the SA content in *PbrMYB14*-overexpressing plants was still significantly higher than in the control (Figure 4d). We further measured the lignin content of leaves using the AcBr method and found that the lignin content in *PbrMYB14*-overexpressing plants was significantly higher than that in control. After inoculation with *A. alternata*, the lignin content in both *PbrMYB14*-overexpressing plants and WT plants was increased, and the lignin content in *PbrMYB14*-overexpressing plants was significantly higher than in WT plants (Figure 4e). Due to the synthesis of lignin and SA through phenylalanine, we tested the activity of phenylalanine ammonia lyase in leaves and found that the phenylalanine ammonia lyase activity in *PbrMYB14*-overexpressing plants were significantly stronger than in WT plants (Figure 4f). After inoculation with *A. alternata*, the phenylalanine ammonia lyase activity in *PbrMYB14*-overexpressing plants were still significantly stronger than that in WT plants. The above results indicate that after pathogen invasion, PbrMYB14 plays a positive role in regulating *A. alternata* resistance by enhancing plant phenylalanine ammonia lyase activity, increasing plant lignin and SA content in pears.

### 2.7. PbrMYB14 Overexpression Hinders A. alternata Invade by Enhancing the Accumulation of Lignin and SA

In order to explore the molecular mechanism of PbrMYB14 in plant resistance to *A. alternata* and identify potential target genes that may be regulated by PbrMYB14, an mRNA sequencing (RNA-seq) analysis was performed on *PbrMYB14*-overexpressing plants and WT plants. After filtering the data, the number of high-quality sequences (Clean Data) of *PbrMYB14*-overexpressing plants and WT plants ranged from 37957960 to 43028888, and the proportions were all higher than 95%. In the base quality analysis, Q20 was greater than 96% and Q30 was greater than 91%. The above data prove that the quality of transcriptome sequencing assembly is high and can be further analyzed.

**Figure 5:**
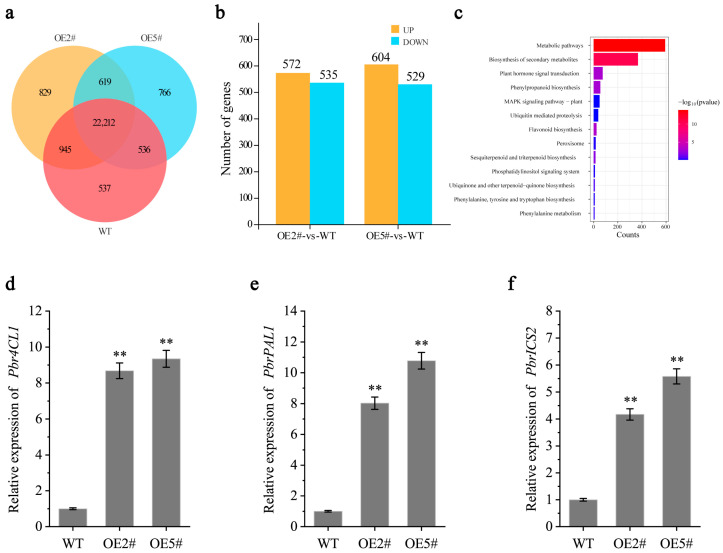
Transcriptome analysis of *PbrMYB14*-overexpressing and WT plants. (**a**) The Venn diagram shows the degree of overlap between WT, OE#2, and OE#5 samples (absolute Log2 [fold change] ≥ 1, q-value ≤ 0.05), with yellow representing OE#2, blue representing OE#5, and red representing WT. (**b**) The number of differentially expressed genes (DEGs) in *PbrMYB14*-overexpressing and WT plants. (**c**) KEGG pathways enriched among the DEGs. (**d**–**f**) The expression levels of *Pbr4CL1*, *PbrPAL1*, and *PbrICS2* in *PbrMYB14*-overexpressing and WT plants. The error bars represent the standard deviation (SD) of three biological replicates. Asterisks indicate significant differences (** *p* < 0.01; based on *t*-test).

In order to explore the disease resistance mechanism of PbrMYB14, a transcriptome correspondence analysis was conducted between *PbrMYB14*-overexpressing and WT plants (Figure 5a). In *PbrMYB14*-overexpressing plants, 515 genes were upregulated and 982 genes were downregulated compared with the WT plants (fold change ≥ 2, FDR < 0.05) (Figure 5b). Through the analysis of disease-related gene pathways, it was found that overexpression of *PbrMYB14* resulted in differential KEGG enrichment in plant disease resistance signaling pathways, MAPK signaling pathways, phenylalanine metabolism, and other pathways (Figure 5c). Among them, SA-related genes such as *PbICS2* and *PbPAL1* were also upregulated (Figure 5d–f).

### 2.8. PbrMYB14 Specifically Binds to the Pbr4CL1 Promoter and Activates the Expression of Pbr4CL1 Genes

**Figure 6:**
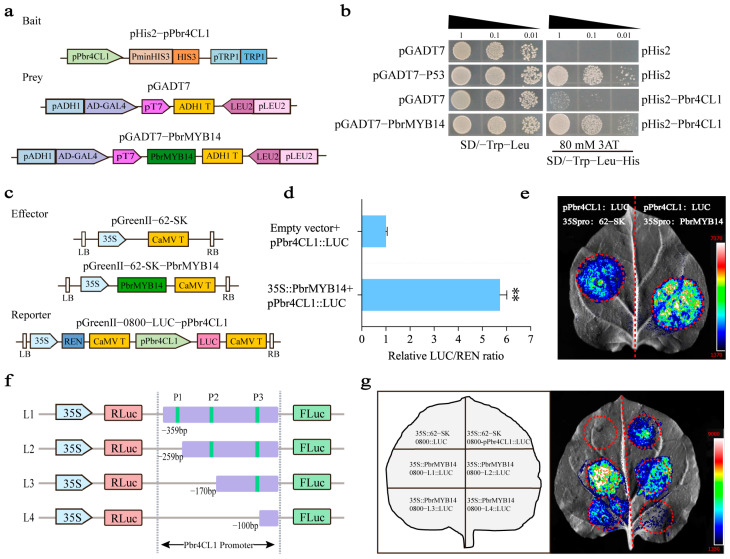
PbrMYB14 directly binds to and activates the promoter of *Pbr4CL1*. (**a**) Schematic diagram of the Y1H vector. *Pbr4CL1* promoters were fused to the HIS2 reporter and PbrMYB14 was fused to GAL4 AD. Transformed yeast cells with the reporter and effector constructs with or without PbrMYB14. (**b**) Y1H assays of PbrMYB14 and *Pbr4CL1* promoter fragments. Transformed yeast cells were grown on synthetic dextrose (SD) media or SD-Trp-His with 3-AT (3-Amino-1, 2, 4-triazole). (**c**) Schematics of the reporter and effector constructs used in the DLR experiment. *Pbr4CL1* promoter was inserted ahead of the *LUC* gene to form a reporter construct, and the empty vector was used as a control. *CaMV35S* promoter-driven PbrMYB14 was used as the effector, and the empty vector was used as the control. LUC, firefly luciferase; REN, Renilla luciferase; 35S, *CaMV35S* promoter; the same below. (**d**) The comparison of luciferase activity. Activation was indicated by the ratio of LUC to REN. The ratio of LUC/REN of the empty vector plus promoter was used for calibration and set to 1. Each value represents the mean ± s.e.m. (*n* = 5); the same below. (**e**) Luciferase activity assay. The red dashed circle represents the injection range. (**f**) Schematic diagram of *Pbr4CL1* promoter truncation in LUC assay. (**g**) LUC activity assay. The red dashed circle represents the injection range. All of the assays were performed least three times with similar results (** *p* < 0.01; based on *t*-test).

The transcriptome results showed that the key gene for lignin synthesis, *Pbr4CL1*, was significantly upregulated in the *PbrMYB14*-overexpressing plants. qRT-PCR processing further confirmed this result (Figure 5c). Therefore, we would like to know if *Pbr4CL1* is a direct target gene of PbrMYB14. To test whether PbrMYB14 binds to the *Pbr4CL1* promoter, we fused the *Pbr4CL1* promoter with the HIS2 reporter, fused PbrMYB14 with GAL4 AD, and tested them in a Y1H assay (Figure 6a). We found that PbrMYB14 indeed interacted with the *Pbr4CL1* promoter, leading to the activation of the HIS2 reporter (Figure 6b).

In the LUC assay, we further validated the binding of PbrMYB14 to the *Pbr4CL1* promoter. We inserted the *Pb4CL1* promoter in front of the *LUC* gene in the pGreenII 0800 vector to form a reporter gene construct (L1), using PbrMYB14 driven by the *CaMV35S* promoter as the effector (Figure 6c). A transient expression analysis of tobacco showed that, compared with the empty vector, the PbrMYB14 promoted the expression of *LUC* driven by the *Pbr4CL1* promoter (Figure 6e). Compared with the corresponding blank control, the instantaneous expression of PbrMYB14 significantly increased the LUC/REN ratio containing the *Pbr4CL1* reporter gene (Figure 6d). The above experimental results indicate that PbrMYB14 has a positive regulatory effect on the *Pbr4CL1* promoter.

Next, we identified three possible binding core sequences by analyzing the *Pbr4CL1* promoter motif (Tabel S2). Therefore, we truncated the *Pbr4CL1* promoter based on the core sequence and constructed them separately into the pGreenII 0800 vector (Figure 6f). Through the Dual-Luciferase assay, this study found that the P1 fragment is the core segment (Figure 6g).

### 2.9. PbrMYB14 Specifically Binds to the PbrPAL1 Promoter and Activates the Expression of PbrPAL1 Genes

**Figure 7:**
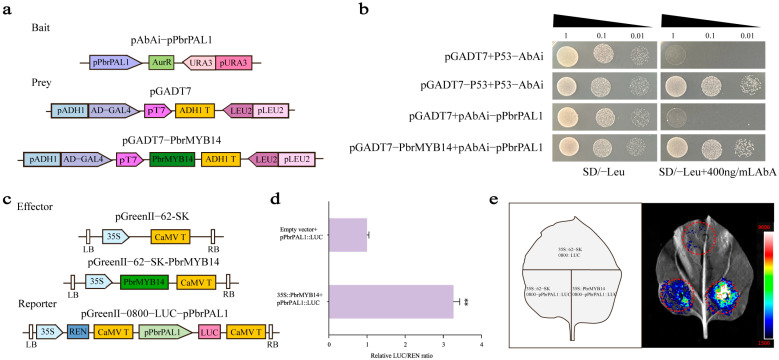
PbrMYB14 directly binds to and activates the promoter of *PbrPAL1.* (**a**) Schematic diagram of the Y1H vector. *PbrPAL1* promoters were fused to the AbAr reporter and PbrMYB14 fused to GAL4 AD. Transformed yeast cells with the reporter and effector constructs with or without PbrMYB14. (**b**) Y1H assays of PbrMYB14 and *PbrPAL1* promoter fragments. (**c**) Schematics of the reporter and effector constructs used in the DLR experiment. *PbrPAL1* promoter was inserted ahead of the *LUC* gene to form a reporter construct, and the empty vector was used as a control. (**d**) The comparison of LUC activity. (**e**) LUC assay. The red dashed circle represents the injection range. All of the assays were performed least three times with similar results (** *p* < 0.01; based on *t*-test).

Previous research results have shown that inoculation of pear leaves with *A. alternata* increases endogenous SA content. We speculate whether PbrMYB14 directly regulates SA synthesis. Therefore, we further analyzed the expression of SA-related genes in the transcriptome. The transcriptome results showed that the key gene for SA synthesis, *PbrPAL1*, was significantly upregulated in the *PbrMYB14*-overexpressing plants; qRT-PCR processing further confirmed this result (Figure 5d). Therefore, we would like to know if *PbrPAL1* is a direct target gene of PbrMYB14. To test whether *PbrMYB14* binds to the *PbrPAL1* promoter, we fused the *PbrPAL1* promoter with the AbAr reporter, fused PbrMYB14 with GAL4 AD, and tested them in a Y1H assay (Figure 7a). We found that PbrMYB14 does indeed interact with the *PbrPAL1* promoter, leading to the activation of the AbAr reporter (Figure 7b).

### 2.10. PbrMYB14 Improves Disease Resistance in Response to SA Signals

**Figure 8:**
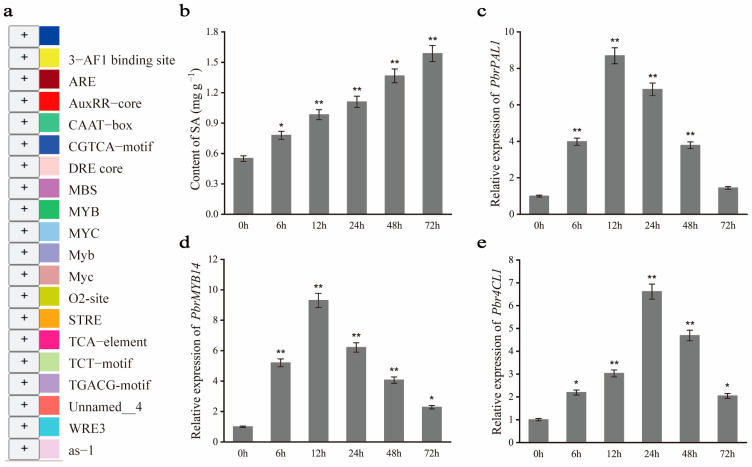
qRT-PCR analysis of SA signals in pear leaves. (**a**) The *cis*-acting regulatory elements of *PbrMYB14* promoter. (**b**) The endogenous SA content in 1-month ‘Xinli No. 7’ test tube seedlings applied with SA. (**c**–**e**) The expression levels of *PbrMYB14*, *PbrPAL1*, and *Pbr4CL1* in 1-month ‘Xinli No. 7’ test tube seedlings applied with SA. The error bars represent the standard deviation (SD) of three biological replicates. Asterisks indicate significant differences (* *p* < 0.05, ** *p* < 0.01; based on *t*-test).

SA is an important plant hormone that activates defense responses in plants and enhances their resistance to pathogens. We analyzed the promoter of *PbrMYB14* (Figure 8a) and discovered acting elements responsive to SA. Moreover, our previous results suggest that SA plays a key role in the fight against *A. alternata*. Notably, the SA content positively regulated the stress response. To further investigate whether exogenous SA application alters the endogenous SA content and expression levels of *PbrMYB14*, *PbrPAL1*, and *Pbr4CL1* in leaves, we conducted experiments using one month old ‘Xinli No. 7’ test tube seedlings. We applied 0.02 mmol/L SA and sampled the plants at 0, 6, 12, 24, 48, and 72 h to assess changes in endogenous SA content and expression profiles of *PbrMYB14*, *PbrPAL1*, and *Pbr4CL1*. Our findings revealed that endogenous SA levels were enhanced (Figure 7b), *PbrMYB14*, *PbrPAL1*, and *Pbr4CL1* expression was significantly upregulated (Figure 7c–e).

### 2.11. Influence of Decreasing PbrPAL1 Expression on Lignin and SA

**Figure 9:**
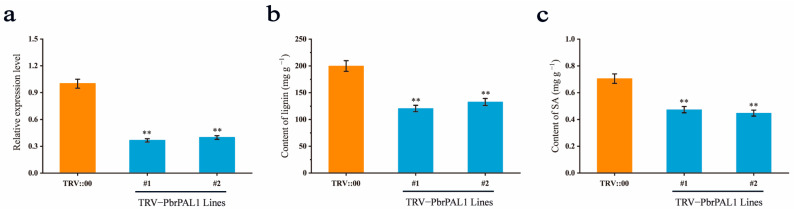
Content of lignin and SA in *PbrPAL1*-silenced plants. (**a**) *PbrPAL1* gene expression levels in *PbrPAL1*-silenced plants. #1 and #2 are both TRV::PbrPAL1 plants, and they are independent of each other. (**b**) HPLC was used to detect the content of SA. (**c**) The AcBr method is used to detect lignin content. The error bars represent the standard deviation (SD) of three biological replicates. Asterisks indicate significant differences (** *p* < 0.01; based on *t*-test).

Previous studies have shown that PAL is involved in the biosynthesis of polyphenolic compounds such as lignin and SA in plants [10]. To demonstrate whether PbrPAL1 is involved in the biosynthesis of lignin and SA in pears, we further evaluated the function of PbrPAL1 through virus-induced gene silencing (VIGS). Leaf cDNAs obtained after 15 days of infection with the empty vector TRV::00 and recombinant vector TRV::PbrPAL1 were used as templates, and pTRV2-F/R was used for PCR-based detection (Table S1). The results showed that the target bands of 206 bp (pTRV2) could be amplified from the ‘Duli’ seedlings inoculated with TRV:00, and bands of 498 bp (TRV::PbrPAL1) could be amplified from the samples inoculated with TRV::PbrPAL1 (Figure S3). These findings indicated that TRV::00 and TRV::PbrPAL1 were successfully inserted and expressed in the genome of ‘Duli’ seedlings. The silencing of the *PbrPAL1* gene in the leaves of ‘Duli’ seedlings was detected by qRT-PCR processing. The leaves were treated with TRV::00 or TRV::PbrPAL1 (with blank control), and after 15 days of treatment, the leaves were collected and RNA was extracted. The results showed that *PbrPAL1* gene expression in the leaves infected with TRV::PbrPAL1 was significantly lower than in the blank control and the leaves infected with TRV::00 (Figure 9a). The results support the idea that *PbrPAL1* was effectively silenced in TRV::PbrPAL1-infected leaves.

To demonstrate whether PbrPAL1 affects the content of SA and lignin, we analyzed lignin and SA accumulation in the TRV::PbrPAL1 and control plants. First, we measured the lignin content of the leaves using the AcBr method and found that the lignin content in the TRV::PbrPAL1 plants was significantly lower than in the control (Figure 9b). To determine the effect of PbrPAL1 on SA levels, we compared SA contents in the TRV::PbrPAL1 plants with the control, and found that the SA content in the TRV::PbrPAL1 plants was significantly lower than that in the control (Figure 9c). These results suggest that PbrPAL1 participates in the biosynthesis of lignin and SA in pear.

## 3. Discussion

*A. alternata*, as a widely existing plant pathogen, has a wide variety of species, diverse infection pathways, and severe symptoms, posing great challenges to agricultural production [29]. The black spot disease caused by the black spot pathogen has resulted in significant economic losses to a wide range of crops, such as pear, tomato, and oat [30,31,32]. Many studies have confirmed that SA metabolism and lignin synthesis play important roles in plant disease resistance [11,33]. SA, as a critical phytohormone, plays a crucial role in the response of plants to pathogen infections and herbivore attacks [34,35]. This triggers systemic acquired resistance (SAR) and allergic reactions (HRs), including disease-related (PR) genes, cascade the amplification of immune signals, local cell death, and defense response [36,37,38]. Lignin not only directly enhances the defense ability of cell walls but also participates in regulating plant immune responses and other disease resistance mechanisms [39,40]. In our study, we isolated the MYB TF and PbrMYB14 and overexpressed it in pear. PbrMYB14 markedly activated SA signaling, along with increased lignin deposition and enhanced resistance to *A. alternata* (Figure 3 and Figure 4). PbrMYB14 simultaneously activated lignin synthesis and SA-mediated resistance pathways, indicating that these two pathways may have a synergistic or complementary relationship in plant defense responses.

MYB TFs are a class of proteins widely present in plants that participate in the regulation of various biological processes, including the synthesis of secondary metabolites, cell differentiation, and response to environmental stress [41,42,43]. The role of MYB TFs is particularly important in plant disease resistance response as they enhance plant defense capabilities by activating lignin, flavonoids, hormone defense signals, and hypersensitivity reactions [44,45,46]. MYB TFs regulate the expression of key lignin synthesis enzyme genes by binding to their promoters, thereby affecting the content and composition of lignin [47]. The increase in lignin enhances the mechanical strength of plant cell walls and improves the plant resistance to pathogenic microorganisms [48]. In addition, MYB TFs interact with other proteins to form regulatory complexes, further affecting lignin synthesis and deposition [49]. MYB TFs also play an important role in the SA signaling pathway. SA is a key endogenous signaling molecule that activate the expression of a series of defense genes and enhance plant disease resistance [50]. MYB TFs regulate the expression of genes related to SA synthesis, affect the level of SA, and thus regulate the disease resistance response of plants [51]. Although some MYB transcription factors have been shown to regulate lignin synthesis genes and resist pathogen invasion and a small number of MYB transcription factors have been shown to participate in the regulation of the salicylic acid signaling pathway, there is very little research to prove whether MYB transcription factors simultaneously regulate both salicylic acid and lignin disease resistance pathways. Our research results prove that PbrMYB14 simultaneously regulates the promoters of the lignin synthesis gene *Pbr4CL1* and the SA synthesis gene *PbrPAL1*, increases the expression levels of Pbr4CL1 and PbrPAL1, and increases the content of lignin and SA, thereby enhancing the resistance of *A. alternata*. Our findings not only enhance our understanding of plant disease resistance mechanisms but also provide new strategies and directions for pear disease resistance breeding.

Previous studies have reported that Os4CL3 is mainly expressed in thick-walled tissue cells near the epidermis, leading to lignification of the sclerenchyma cell [22]. The inhibition of *Os4CL3* expression significantly resulted in a decrease in total lignin content and each lignin subunit, with slight variations in the proportion of individual subunits [52]. According to reports, *Arabidopsis* MYB15 is the closest *Arabidopsis* homolog to OsMYB30, which regulates lignin biosynthesis [21]. One possibility is that OsMYB30 also regulates lignin biosynthesis-related genes other than *Os4CL3* and *Os4CL5*, such as *PAL1*, *C4H1*, *C3H*, and *COMT* genes. This is consistent with the results of this study. In this study, overexpression of *PbrMYB14* resulted in the upregulation of lignin and flavonoid synthesis-related genes such as *COMT*, *CCOAOMT*, and *HCT* in the transcriptome analysis. This indicates that PbrMYB14 not only regulates the key lignin synthesis gene *Pbr4CL1* but also directly or indirectly regulates other lignin synthesis-related genes. In previous reports, MYB TFs have been shown to bind to AC-rich motifs. In our study, we identified three AC-rich sequences through analysis of the *Pbr4CL1* promoter, consistent with previous research results. Our LUC promoter truncation assay showed that PbrMYB14 bind to L1 fragment sequences rich in AC elements (Figure 6g).

Previous studies have shown that both lignin and SA respond to the pathogens invasion [46,53], but it is not clear whether there is a connection between the two disease resistance pathways. In this study, we found a MYB TF that upregulated lignin and SA content in pears after overexpression of *PbrMYB14*. This indicates that PbrMYB14 positively regulates both lignin and SA resistance pathways, enhancing pear resistance to black spot disease, connecting these two disease resistance pathways closely together. Previous studies have shown that OsMYB30 resists pathogen invasion by positively regulating *Os4CL3* and increasing lignin content [22]. Therefore, we verified through Y1H and LUC assays that PbrMYB14 in pear binds to *Pbr4CL1* and positively regulates lignin synthesis (Figure 6). SA is synthesized through two pathways, PAL and ICS [54,55]. In recent studies, it has been pointed out that the phenylalanine ammonia lyase pathway in *Arabidopsis* synthesizes the isomer 4-HBA of SA, not SA [56]. This conclusion overturns the classical theory that 10% of SA is synthesized through the phenylalanine pathway. However, in our study, reducing the expression of *PbrPAL1* did indeed lead to a decrease in endogenous SA in pears, so we believe that SA is synthesized through the phenylalanine ammonia lyase pathway in pears. We speculate that the different synthetic pathways of SA may be due to species differences.

By analyzing the transcriptome data of *PbrMYB14*-overexpressing plants, we found that the key gene for SA synthesis, *PbrICS2*, was also induced to upregulate. The same results were verified by qRT-PCR (Figure 5e). Therefore, we want to know if PbrMYB14 directly regulates *PbrICS2*. Through Y1H and LUC experiments, it has been proven that PbrMYB14 does not directly regulate *PbrICS2* (Figure S4). Plant disease resistance is a complex process, and PbrMYB14 may upregulate the expression of *PbrICS2* through a series of complex cascade reactions. Previous studies have shown that WRKY TFs regulate the expression of *ICS*, while MYB TFs and WRKY TFs interact with each other at multiple levels, including functional complementarity, signal pathway crossing, protein interaction, and expression regulation, to jointly regulate plant disease resistance responses. This discovery provides new ideas for further research on the relationship between PbrMYB14 and the pathway of ICS in the future.

**Figure 10:**
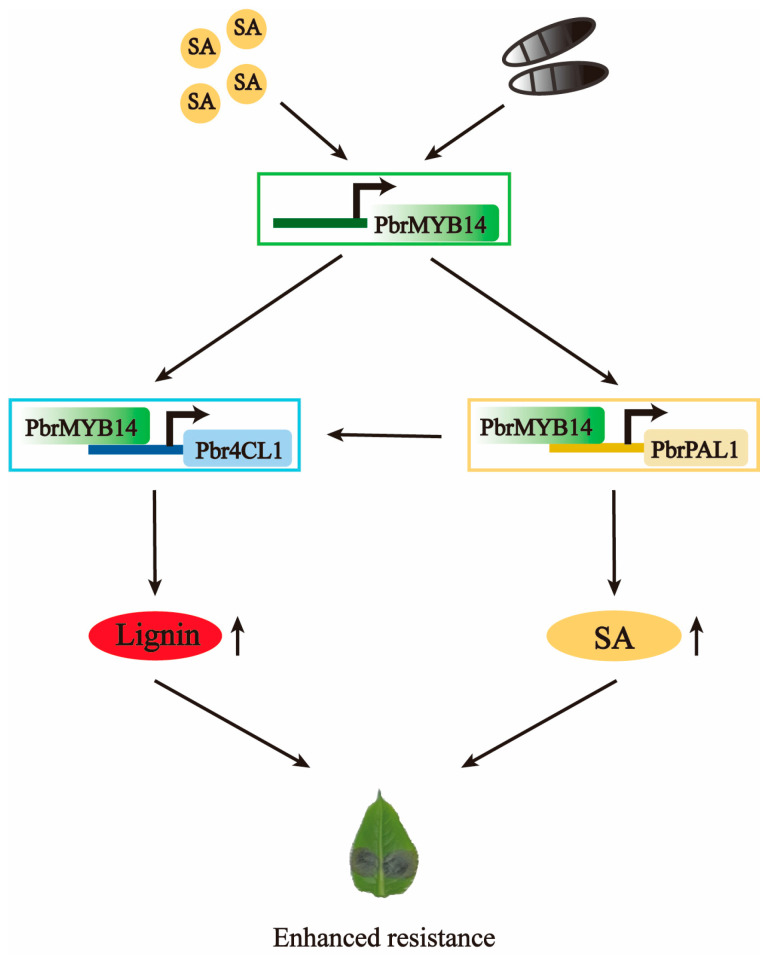
Hypothetical model of PbrMYB14 role in the development of pear black spots. *A. alternata* infection and application of exogenous SA both results in the upregulation of *PbrMYB14* expression. On the one hand, PbrMYB14 promotes the expression of the *Pbr4CL1* gene by binding to the promoter of the lignin synthesis gene *Pbr4CL1*, and on the other hand, it promotes the expression of the *PbrPAL1* gene by binding to the promoter of the SA synthesis-related gene *PbrPAL1*. These changes will lead to an increase in lignin and SA content in pears, thereby enhancing their resistance to *A.alternata*. Solid lines represent the determined regulatory effect, and arrows represent the facilitated effect.

In summary, our results indicate that exogenous SA treatment and *A. alternata* infection enhance the expression of *PbrMYB14*. In order to elucidate the possible resistance mechanism mediated by PbrMYB14, we conducted molecular, genetic, and biochemical analyses and described the role of the the PbrMYB14-PbrPAL1-Pbr4CL1 model in combating *A. alternata*. We demonstrate that PbrMYB14 positively regulates resistance to *A. alternata*. In *PbrMYB14*-overexpressing plants, the production of PbrMYB14 upregulates the expression of *PbrPAL1* and *Pbr4CL1*, enhances the physical barrier and endogenous SA content through lignin deposition, activates the plant disease resistance and defense response, and jointly promotes pear resistance to *A. alternata* (Figure 10).

## 4. Materials and Methods

### 4.1. Plant Materials and A. alternata

Take the stem segments (approximately 1–2 cm) of the ‘Xinli No. 7’ from the experimental park of Hebei Agricultural University as explants and culture and propagate them for inoculation with *A. alternata* and SA treatment. Using ‘Duli’ seedlings as experimental materials, VIGS processing was conducted to study gene function.

### 4.2. Pear Leaf Inoculation

The spore suspension was prepared by using an *A. alternata* strain cultured in dark at 25 °C for about 15 days on PDA medium. The mycelium was scraped and washed with sterile water, and the obtained spore suspension was filtered with four layers of gauze. A blood cell counting plate was used to prepare a concentration of 5 × 10^6^ cfu/mL. Take 5.0 μL of suspension of conidia and needle-inoculate it onto pear leaves; sterile water was used as a blank control. Inoculate ten pear test tube seedlings for each treatment and inoculate three leaves per pear test tube seedling. The storage conditions for the test tube seedlings were: light intensity of 2500~4000 Lx, light cycle of 16 h/8 h, temperature of 25 °C, and relative humidity 60%.

### 4.3. Histochemical Staining and Determination of Total Lignin Content

To observe the lignin deposition in wild-type and transgenic plants, hand-cut cross-sections from the stems of ‘Xinli No. 7’ were prepared and stained with phloroglucinol solution according to a slightly modified version of the previous method [57]. In brief, the sections were dipped in a phloroglucinol solution (3% *w*/*v* phloroglucinol in 95% ethanol) for 15 min, then quickly transferred into 18% HCl for 5 min, and immediately photographed under a microscope with bright field illumination. The total lignin content was determined by the acetyl bromide method. The dried leaves were ground into powder, and three biological replicates were determined for each treatment. The specific steps were carried out according to the instructions of the Grice biological lignin assay kit (Suzhou, China).

### 4.4. Analyses of SA Contents

After freezing and grinding the pear leaves in liquid nitrogen, weigh 0.5 g and transfer it to a 10 mL centrifuge tube. Add 0.5 mL of 5% trichloroacetic acid and dilute to 3 mL with ultrapure water. Then, add 2 mL of ether, mix well, and extract at 4 °C for 12 h. Centrifuge at 4 °C and 8000 rpm for 10 min, take the upper phase, repeat the extraction three times, and then create up to 5 mL of solution with ether. After nitrogen blowing and drying, add 1 mL of methanol to dissolve, which is the free-state SA sample to be tested. Add 0.5 mL of 18.5% HCl to the lower phase, conduct a water bath at 80 °C for 1 h, cool and add 2 mL of ether, mix well, and let the solution stand. Take the upper phase, repeat the extraction three times, and create up to 5 mL of solution with ether. Blow dry with nitrogen and dissolve in 1 mL of methanol. This is the bound-state SA sample to be tested. All samples were injected into the injection bottle through a 0.22 µm filter membrane for HPLC analysis. The mobile phase and measurement conditions are A: methanol, B: 65% acetonitrile solution, A:B = 1:1, flow rate 1 mL/minute, and injection volume 20 μL [58].

### 4.5. Determination of Phenylalanine Ammonia Lyase Activity

We measured PAL activity using the PAL test kit (A137-1-1) from Nanjing Jiancheng Bioengineering Research Institute (Nanjing, China). Accurately weigh 0.1 g of the sample, strictly follow the instructions for measurement, use a spectrophotometer to measure PAL activity, and then calculate PAL activity according to the formula shown in the instructions.

### 4.6. Subcellular Localization Analysis

Using the recombinant T plasmid as a template, the intact coding sequence for *PbrMYB14* was amplified by using specific primers and then fused to a *35S:*GFP vector (*Apa*I). The recombinant plasmid (*35S:PbrMYB14*-GFP) was introduced into the *A. tumefaciens* strain (GV3101) and mixed with nuclear localization maker NLS-mcherry for transient expression in 4-week-old tobacco (*N. benthamiana*) leaves. In addition, *A. tumefaciens* harboring a *35S:*GFP plasmid was transiently expressed in tobacco leaves as the control. The GFP signal was visualized with fluorescence microscope 48 h post-infiltration [59].

### 4.7. Transcriptional Activation Activity Assay

The full-length CDS of *PbrMYB14* was amplified by PCR using the primers pGBKT7-PbrMYB14 F and pGBKT7-PbrMYB14 R and was ligated downstream to the GAL4 DNA-binding domain (GAL4BD) in the pGBKT7 vector (Vazyme, C112, Nanjing, China) by homologous recombination. The fusion construct and empty vector control were transformed into the yeast strain Y2HGold and plated on synthetic dropout medium without tryptophan (SDO). Then, the transformed cells were cultured on selective SDO medium supplemented with 40 μg/mL X-α-gal (5-bromo-4-chloro-3-indolyl-α-D-galactoside) and 200 ng/mL AbA (aureobasidin A) (SDO/X/A) to assess the transcriptional activation potential of PbrMYB14 for the *MEL1* and *AUR1* reporter genes [60].

### 4.8. Plasmid Construction and Genetic Transformation of ‘Duli’ Seeds

The ‘Duli’ seeds were washed under running water for 8 h, treated with 0.1% HgCl_2_ in ultra clean bench for 8 min, treated with 75% anhydrous ethanol for 2 min, and finally washed with sterile distilled water 5 times. After disinfection, the inner seed coat was removed on an ultra clean bench; the germ, cotyledon, and radicle were cut off; and two cotyledons were removed and horizontally inoculated in the regeneration medium (Nitsch–Nitsch medium 1969, 5 mg/L 6-BA, 0.05 mg/L NAA, 30 g/L sucrose, 6 g/L agar; the pH value is 5.8). The cotyledons were used for transformation after 7 days of dark culture. The *A. tumefaciens* strain, EHA105, containing the overexpressed recombinant vectors, was used to transform the cotyledons. The cotyledons were inoculated in *Agrobacterium* suspension (OD600, 0.7) for 8 min, and the infected cotyledons were placed on co-culture medium and cultured in dark for 2 days. After co-culture, the cotyledons were transferred to the screening medium (Hyg 7 mg/L, Temetin 200 mg/L) and cultured under light to induce adventitious buds. After the resistant buds grew to about 1cm, they were cut off and cultured on the subculture medium containing Temetin 200 mg/L. All cultures described above were maintained in the growth chamber at 25 °C [61].

### 4.9. RNA-Seq and Analysis

Take 0.5 g of mixed leaves from wild-type and overexpressing plants for RNA sequencing (RNA Seq) experiments. The cDNA libraries were sequenced on the Illumina sequencing platform by Genedenovo Biotechnology Co, Ltd. (Guangzhou, China). The Nr, Nt, Pfam, KOG/COG, Swiss-Prot, KO, and GO databases were used to annotate gene ontology. Gene expression levels were indicated by the FPKM (fragments per kilobase of exon per million fragments mapped) values. Fold change ≥2 and false discovery rate (FDR) < 0.01 were used to screen and identify differentially expressed genes (DEGs) between samples. The Gene Ontology (GO) enrichment analysis and the Kyoto Encyclopedia of Genes and Genomes (KEGG) pathway analyses were performed using OmicShare tools (www.omicshare.com/tools, URL (accessed on 16 May 2024)).

### 4.10. RNA Extraction and Quantitative Real-Time PCR (qRT-PCR)

The total RNA was isolated from test tube seedlings of ‘Xinli No. 7’ using the RNA prep Pure Plant Kit according to the manufacturer’s protocol (Omega Bio-Tek, Norcross, GA, USA). The first-strand cDNA was synthesized from 1 µg of total RNA using the First-Strand cDNA Synthesis Kit (Yeasen, Shanghai, China). The transcript levels of the identified genes were analyzed by qRT-PCR using the above-synthesized cDNAs as templates, Hieff UNICON^®^ Universal Blue QPCR SYBR Green Master Mix (Yeasen, Shanghai, China), and gene-specific primers (Table S1) in a 20 µL reaction mix. The reaction was run on a Step-Two Plus real-time PCR system (Mastercyler ep realplex4, Eppendorf AG, Hamburg, Germany). The qRT-PCR procedure was performed as follows: 30 s of template pre-denaturation at 95 °C, 3 s of template denaturation at 95 °C, 10 s of primer annealing at 60 °C for 40 cycles, followed by the melting curve analysis. The normalized expression level of each gene was calculated using the 2^−∆∆CT^ method; *PbActin* was used as the reference gene. At least three replicates were included in each experiment, and experiments were repeated three times.

### 4.11. One-Hybrid Assays in Yeast

The full-length cDNA sequence of *PbrMYB14* was amplified and fused in frame with the GAL4 activation domain in pGADT7, forming pGADT7-PbrMYB14. Then, the fusion construct was co-transformed with the reporter vector (pHIS2-promoter of *Pbr4CL1*) into Y187 yeast cells. The sequences of the primers are listed in Supplementary Materials Table S1. The empty vector pGADT7 and the pHIS2-promoter were co-transformed as the control for mating experiments. DNA–Protein interactions were determined by the growth of the transformants on nutrient-deficient medium containing different concentrations of 3-amino-1,2,4-triazole (3-AT) [22].

### 4.12. Dual-Luciferase Reporter Assay

The *Pbr4CL1* promoter (a 1667 bp fragment upstream of the start codon) and *PbrPAL1* promoter (a 1091 bp fragment upstream of the start codon) were amplified by PCR and inserted ahead of the *LUC* gene in the pGreenII 0800 vector to form a promoter-LUC reporter construct. The full-length CDS of *PbrMYB14* was fused downstream to the *CaMV35S* promoter in the pGreenII 62-sk vector to generate an effector construct. All of the constructs and the helper plasmid pSoupp19 were introduced into *Agrobacterium tumefaciens* strain GV3101 and agro-infiltrated into tobacco leaves for the transient gene expression assay [46]. LUC and REN activities were quantified using the Dual-Luciferase^®^ Reporter Assay System (Promega, Madison, WI, USA). The Dual-Luciferase^®^ Reporter Assay System (Promega, USA) was used to examine the firefly and Renilla luminescence according to the manufacturer’s instructions. The promoter activity was expressed as a ratio of LUC to REN activity.

### 4.13. Virus-Induced Gene Silencing

To specifically silence the *PbrPAL1* gene, we amplified a 498 bp fragment of the gene and cloned it into the pTRV2 vector. The correct recombinant vector was verified by PCR and sequencing.

The pTRV1, pTRV2, and pTRV2-*PbrPAL1* plasmids were transformed into *Agrobacterium tumefaciens* GV3101. Transfer the *Agrobacterium*-carrying recombinant vector into LB liquid medium (including 50 µg/mL Kan, 100 µg/mL Rif, and 200 µM Acetosyringone (AS)) and culture at 28 °C for 2 days at a rate of 200 r min^−1^. When the OD600 of the bacterial liquid is 1, collect and resuspend the solution (10 mmol/L MES, 10 mmol/L MgCl_2_, 150 µM AS, and aseptic water as solvent). The OD600 of the suspension was adjusted to 1. Use GV3101-pTRV2-PbrPAL1 mixed with GV3101-pTRV1 at a 1:1 ratio and mix GV3101-pTRV2 with GV3101-pTRV1 at a 1:1 ratio as a negative control. Allow the mixture to rest for 2–4 h at room temperature in darkness [62].

Select 30 days old ‘Duli’ seedlings, inject three new leaves with heavy suspension, and then cultivate in the dark for 24 h before turning to light. Afterwards, perform normal field management procedures on the treated plants and control plants.

### 4.14. Quantification and Statistical Analysis

Statistical analyses were performed using SPSS 13.0 software (SPSS Inc., Chicago, IL, USA). All values are presented as the mean ± SD, and the numbers (n) of samples are indicated in the data legend. Normality of data and homogeneity of variances were determined using the Shapiro–Wilk and the Levene tests, respectively. Statistically significant differences between control and experimental groups were determined by one-way ANOVA.

## 5. Conclusions

In conclusion, we have successfully cloned the coding sequence of the *PbrMYB14* gene. The phylogenetic analysis and amino acid sequence alignment indicate that PbrMYB14 shares high homology with *Arabidopsis* SG2-type R2R3-MYB transcription factor AtMYB15, suggesting that PbrMYB14 is an SG2-type R2R3-MYB transcription factor. The subcellular localization results of this study indicate that the fusion expression vector GFP fluorescent protein material exists in the nucleus, confirming that the metabolites of the *PbrMYB14* gene were expressed in the nucleus. This suggests that MYB14 protein mainly functions in the nucleus. The transcriptional activation activity assay demonstrated that PbrMYB14 is a transcriptional activator. Through *Agrobacterium*-mediated genetic transformation of ‘Duli’ cotyledons, *PbrMYB14*-overexpressing plants were obtained. Compared with WT, the *PbrMYB14*-overexpressing plants showed increased PAL activity, lignin content, and SA content after invasion by *A. alternata*. This indicates that lignin and SA play a synergistic role in pear resistance to *A. alternata*. In order to further explore the molecular mechanisms connecting these pathways, we screened the upregulated lignin synthesis gene *Pbr4CL1* and SA synthesis gene *PbrPAL1* induced by PbrMYB14 through transcriptome sequencing. Through one-hybrid assays in yeast and Dual-Luciferase assays, it was demonstrated that PbrMYB14 positively regulates the expression of lignin synthesis gene *Pbr4CL1* and SA synthesis gene *PbrPAL1*, increasing the endogenous SA and lignin content in pears, thereby enhancing their resistance to *A. alternata*.

In future research, we will prioritize investigating whether *PbrMYB14*-overexpressing plants have broad-spectrum resistance, that is, whether they resist their fungal and bacterial diseases. After solving this problem, we will further investigate whether the growth, development, and fruit quality of *PbrMYB14*-overexpressing plants were affected in the field. We believe that in the future, we will rapidly reproduce a large number of plants with high disease resistance and good genetic stability through genetic improvement and tissue culture technology and apply our research results to commercial pear cultivation.

## Data Availability

Data are contained within the article and Supplementary Materials.

## Supplementary Materials

The following supporting information can be downloaded at: https://www.mdpi.com/article/10.3390/ijms26030972/s1.
