# Supplementary material for: PbrMYB14 Enhances Pear Resistance to Alternaria alternata by Regulating Genes in Lignin and Salicylic Acid Biosynthesis Pathways
Source: Int J Mol Sci. 2025 Jan 24;26(3):972. doi: 10.3390/ijms26030972 (PMC11817059; doi:10.3390/ijms26030972)
Supplement: Supplementary file 1 [file ijms-26-00972-s001.zip › Supplementary Figures and Tables.pdf]

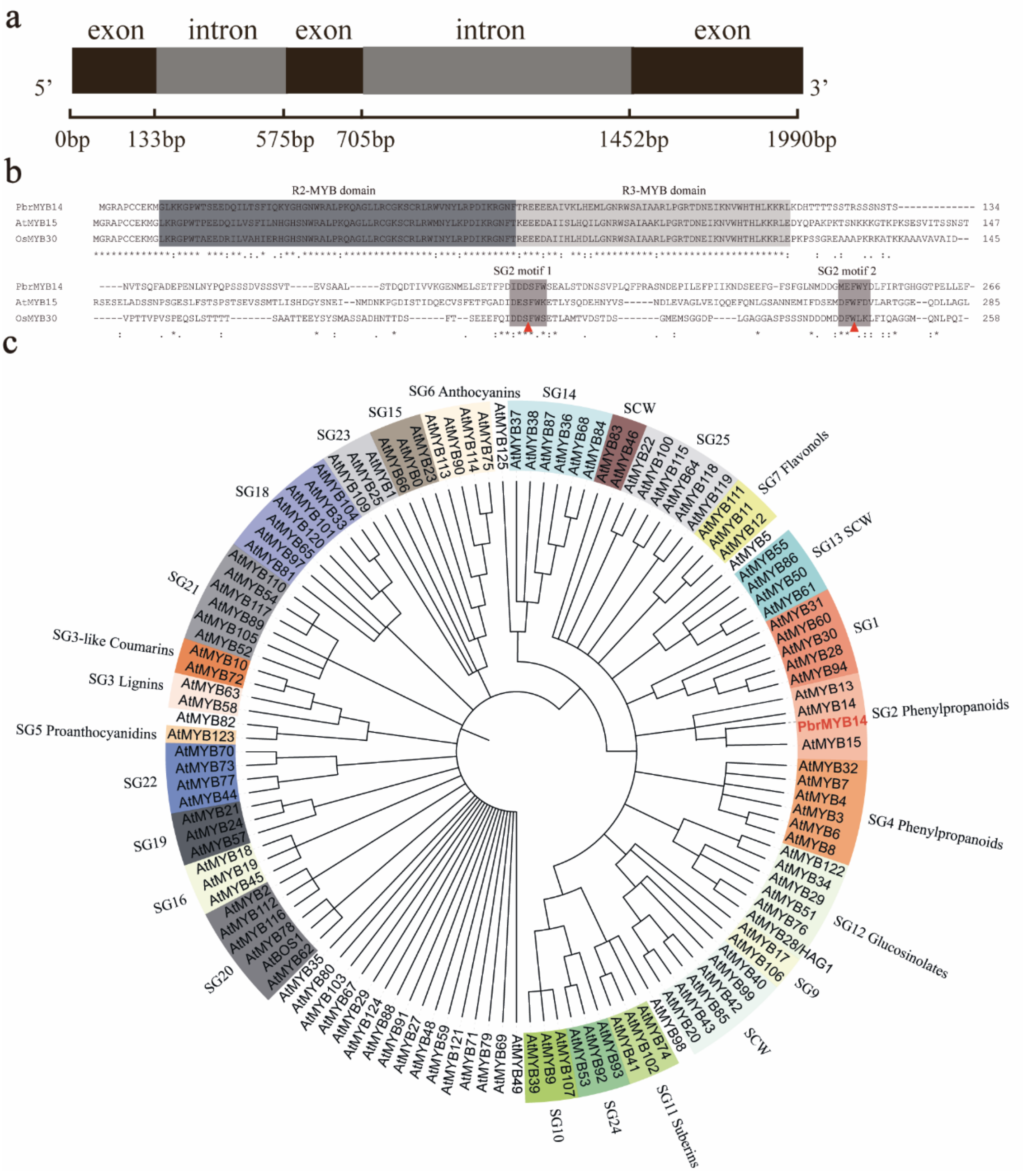

Figure S1. Bioinformatics analysis of PbrMYB14. (a) Structure of the *PbrMYB14* sequence. (b) Multiple alignment of the deduced PbrMYB14 amino acid sequences with its homologs. Note: The grey box represents the conserved domain of R2R3-MYB; the red triangle indicates the SG2SG3 motif. (c) Phylogenetic analysis based on the amino acid sequences of PbrMYB14 in pear and MYB family genes in *Arabidopsis*.

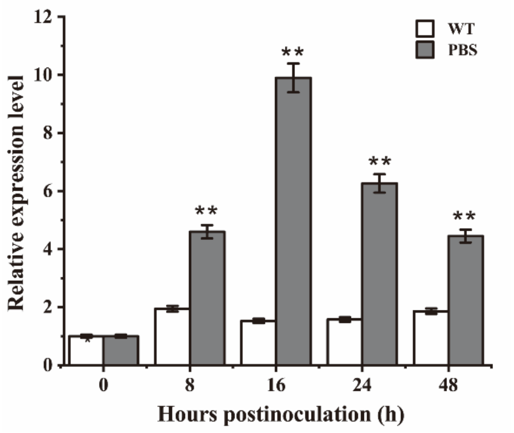

Figure S2. Expression patterns of *PbrMYB14* under infection with *A. alternata*.

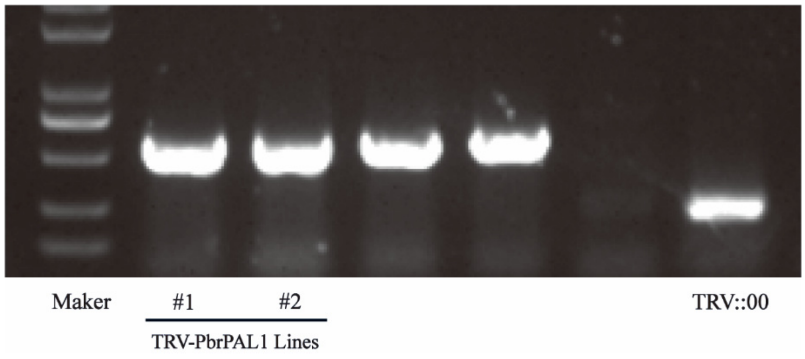

Figure S3. The PCR identification of TRV2 in *PbrPAL1*-silenced plants.

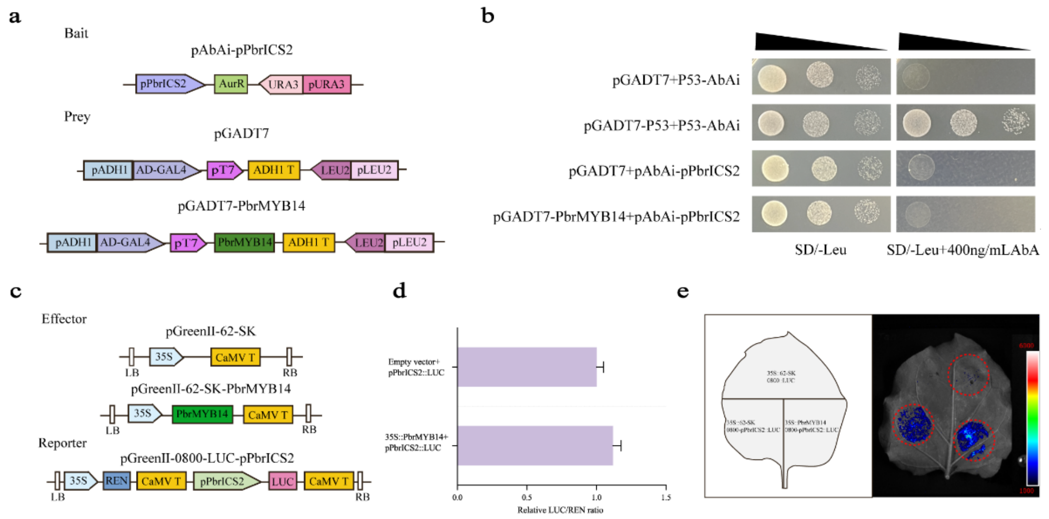

Figure S4. (a) Schematic diagram of Y1H vector. *PbrICS2* promoters was fused to the AbAr reporter and PbrMYB14 fused to GAL4 AD. Yeast cells-transformed with the reporter and effector constructs with or without PbrMYB14. (b) Y1H assays of PbrMYB14 and *PbrICS2* promoter fragments. (c) Schematics of the reporter and effector constructs used in the DLR experiment. *PbrPAL1* promoter was inserted ahead of the *LUC* gene to form a reporter construct, and the empty vector was used as a control. (d) Luciferase activity assay. (e) The comparison of luciferase activity.

Table 1. Sequence of primers used for gene cloning and qRT-PCR.

Primer	Sequence (5'-3')
Peasy-PbrMYB14 F	TACTGCTGTATTCTTAGC
Peasy-PbrMYB14 R	CCAACCTAGCATTTGTTAAC
Peasy-Pbr4CL1 F	CATCCAGTTGTATATGAAGAACC
Peasy-Pbr4CL1 R	GCCCTTCCTTCAAAATGGCAG
Peasy-PbrPAL1 F	ATGGAGGCGGAAACCATC
Peasy-PbrPAL1 R	CTAACAGATAGGAAGAGG
p35S-GFP-PbrMYB14 F	CGACGACAAGACCGTCACCATGGGGAGAGCTCCTTGCTG
p35S-GFP-PbrMYB14 R	GAGGAGAAGAGCCGTCGTCAAAATTCTGGTAATTCTGGCG
pGBKT7-PbrMYB14 F	GCCATGGAGGCCGAATTCATGGGGAGAGCTCCTTGCTG
pGBKT7-PbrMYB14 R	CTGCAGGTCGACGGATCCTCAAAATTCTGGTAATTCTGG
pGADT7-PbrMYB14 F	GGGGACAAGTTTGTACAAAAAAGCAGGCTTCATGGGGAGAGC TCCTTGCTG
pGADT7-PbrMYB14 R	GGGGACCACTTTGTACAAGAAAGCTGGGTCAAATTCTGGTAAT TCTGGCGTTCC
pHis2-pPbr4CL1 F	CGACGACAAGACCGTCACCCCTCAACCTTGTTCTCCCAC
pHis2-pPbr4CL1 R	GAGGAGAAGAGCCGTCGCTCTTGGAAAAATGGGATTATCC
pAbAi-pPbrPAL1 F	GAATTCGAGCTCGGTACCCCTTTGATTCTTAATATGACTATG
pAbAi-pPbrPAL1 R	CATGCCTCGAGGTCGACGTTAATTACGAAAAACTGAAAAC
pAbAi-pPbrICS2 F	GAATTCGAGCTCGGTACCGCAGTGAGACTAATGAGACGC
pAbAi-pPbrICS2 R	CATGCCTCGAGGTCGACTTTGGTGGGAAATGTGACAC
pGreenII-62-sk-PbrMYB14 F	GCTCTAGAACTAGTGGATCCATGGGGAGAGCTCCTTGCTG
pGreenII-62-sk-PbrMYB14 R	ATAAGCTTGATATCGAATTCTCAAAATTCTGGTAATTCTGG
pGreenII-0800-pPbr4CL1 F	GGTATCGATAAGCTTGCAGCCTCTTCAAAGTACGCGAC
pGreenII-0800-pPbr4CL1 R	CGCTCTAGAACTAGTCTCTTGGAAAAATGGGATTATCC
pGreenII-0800-pPbrPAL1 F	GGTATCGATAAGCTTCCTTTGATTCTTAATATGACTATG
pGreenII-0800-pPbrPAL1 R	CGCTCTAGAACTAGTGTTAATTACGAAAAACTGAAAAC
pGreenII-0800-pPbrICS2 F	GGTATCGATAAGCTTGCAGTGAGACTAATGAGACGC
pGreenII-0800-pPbrICS2 R	CGCTCTAGAACTAGTTTTGGTGGGAAATGTGACAC
pTRV2- PbrPAL1 F	ACGTGGATCCCGGCGGATTCACTAAAGGG
pTRV2- PbrPAL1 R	CGCGTGAATCCTGAAGGGCAGCGCCTTGC
pTRV2 F	TTATTACGGACGAGTGGACTTAGAT
pTRV2 R	TCAAGATCAGTCGAGAATGTCAATC
Action F	TTGGTATGGGTCAGAAGG
Action R	CTGTGAGCAGAACTGGGTG
qPbrMYB14 F	GCTGCATTAAGTACCGATC
qPbrMYB14 R	GAAATTGTAATGGGACGCTG
qPbr4CL1 F	GATACTGTGAGAGCCAAGTT
qPbr4CL1 R	GATCTGGTCACCTCTAATGC
qPbrPAL1 F	TCCAAGCTATCTGCCAGGGA
qPbrPAL1 R	GCAGCACTCAAGCCGTAACA
qPbrICS2 F	TTCATGGATCCCGAAGCAGC
qPbrICS2 R	AAACAGCGCGGAAGGAATTG
qPbrNPR1 F	AGGATACACAGGGCTTTGGATTCG
qPbrNPR1 R	AACACAGTGTACCCCCTCGGATTC
qPbrPR1 F	GCAGCAGTAGGCGTTGGTCCCT
qPbrPR1 R	CCAGTGCTCATGGCAAGGTTTT
pGreenII-0800-pPbr4CL1-L1 F	GGTATCGATAAGCTTATCGTCAGCCAGTGGTTG
pGreenII-0800-pPbr4CL1-L2 F	GGTATCGATAAGCTTACTCATCCCTTCCGCCT
pGreenII-0800-pPbr4CL1-L3 F	GGTATCGATAAGCTTCCCAAACAATTCCATAATCC

pGreenII-0800-pPbr4CL1-L4 F	GGTATCGATAAGCTTCTATTGACCTCCCCTCTTC

[1] Restriction sites/recombination sites are underlined.

Table 2. *Pbr4CL1* binding site sequence

**Binding site**	**Sequence (5'-3')**
P1	CAAACCCACTTTCACCAACCA
P2	GTCACCTATCACCACCAACCC
P3	TTCCCTCCCCCCCACCAACCA
